# Design Principles for Alloy‐Anode‐Based Low‐Stack‐Pressure Solid‐State Batteries

**DOI:** 10.1002/advs.76946

**Published:** 2026-08-03

**Authors:** Yuping Huang, Zhe‐Tao Sun, Xinyu Yu, Shiwei Chen, Chen Su, Jia Li, Shou‐Hang Bo, Hong Zhu

**Affiliations:** ^1^ Global College Shanghai Jiao Tong University Shanghai China; ^2^ Future Battery Research Center Global Institute of Future Technology Shanghai Jiao Tong University Shanghai China; ^3^ Aviation & Robot Battery Technology Center Contemporary Amperex Technology Co., Ltd. Ningde China; ^4^ School of Chemistry and Chemical Engineering Shanghai Jiao Tong University Shanghai China

**Keywords:** design principles, low stack pressure, solid‐state batteries

## Abstract

Solid‐state batteries with Li alloy anodes offer enhanced safety and energy density. However, many studies still rely on high stack pressures, while low stack pressure operation is essential for practical application. This work establishes a general electro‐chemo‐mechanical framework that enables rational pairing of alloy anodes and solid electrolytes by predicting the critical stack pressure required for interfacial stability under practical operating conditions. Based on thermodynamic and mechanical factors, our findings identified three design principles for achieving low stack pressure: (1) applying highly conductive and mechanically compliant solid electrolytes; (2) using Li‐rich and hard alloy anodes; and (3) optimizing external conditions through smooth interfaces, low applied current densities, and elevated temperatures. Experimental results demonstrate that the LiAl alloy paired with Li_6_PS_5_Cl electrolyte exhibits stable cycling performance and smooth interfacial morphology above the critical stack pressure, while poor performance with rough morphology has been observed below it. These findings provide key principles for achieving low stack pressure in solid‐state batteries.

## Graphical abstract

Design principles for matching alloy anodes with solid electrolytes to achieve low‐pressure solid‐state batteries are identified through electro‐chemo‐mechanical modeling, which reveals the critical stack pressure for interfacial stability, and validated experimentally. Softer, highly ion‐conductive solid electrolytes, Li‐rich harder alloy anodes, and low‐roughness interfaces enable stable cycling and suppress dendrite growth, offering a practical route toward low‐pressure solid‐state batteries.

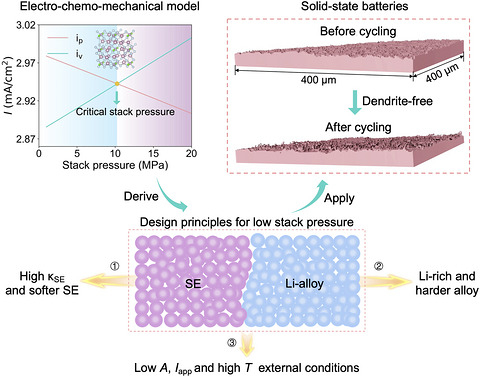

## 1 Introduction

Solid‐state batteries (SSBs), with their superior safety and higher power densities, are promising alternatives to conventional lithium‐ion batteries (LIBs) [1, 2, 3]. These advantages stem primarily from the usage of solid electrolytes (SEs), which reduce electrochemical crosstalk and enable rapid charging due to their high lithium‐ion transference numbers [4, 5]. To achieve higher energy densities exceeding 400 Wh/kg [6, 7], the application of high‐capacity metallic anodes such as lithium (Li) metal or its alloys is essential. Compared to pure Li, Li alloy anodes, e.g Li‐In, Li‐Mg, Li‐Si and Li‐Ag [8, 9, 10, 11], demonstrated enhanced diffusivity and lower SE reactivity, effectively mitigating dendrite growth in SSBs [12, 13, 14].

Recent experimental studies reveal that stack pressure plays a critical role in SSBs’ performance by improving the alloy–SE interfacial contact and hence reducing the interfacial resistance [15, 16, 17]. Stack‐pressure‐dependent anode structural evolution has been observed. For instance, stack pressure regulated morphology enables compact Li deposition and efficient strippings, significantly boosting Coulombic efficiencies [18]. Furthermore, evidence suggests that a stack pressure of around 350 kPa [19] is necessary to maintain dense Li deposits in liquid electrolytes. The prevailing literature reports that stable cycling of alloy‐anode SSBs typically necessitates substantial stack pressure ranging from 20 to 250 MPa [20, 21, 22, 23, 24, 25, 26, 27]. However, such extreme mechanical loading presents significant commercialization challenges, as practical cells (e.g., 4 × 4 cm^2^ area) would demand compressive forces surpassing 10 tonnes (∼61 MPa). Achieving such extreme stack pressure in a commercially viable battery enclosure would require heavy constructions [28], inevitably compromising the energy density at the pack level and posing substantial challenges for large‐scale production and application. Previous studies have commonly focused on stack pressures around 2 MPa, which is also adopted here as a representative reference value. Consequently, operation under low stack pressure (<2 MPa) has become a widely used research paradigm in the field [29, 30, 31]. To reduce the stack pressure of SSBs, previous studies have explored various strategies, including the development of composite anodes to mitigate its volume expansion [32], the usage of viscoelastic inorganic SEs to enhance interfacial contact [33] and reducing the charge cut‐off voltage to limit electrode volume changes [34]. To realize low stack pressure operation in SSBs, it is essential to establish comprehensive design principles from the perspective of materials selection as well as the service conditions.

Chemo‐mechanical or electro‐chemo‐mechanical coupling analysis is essential to evaluate the interface stability between SEs and anodes in SSBs, which governs dendrite formations [35, 36]. A chemo‐mechanical model incorporating linear elasticity has revealed that polymer electrolytes should have a shear modulus at least twice that of Li metal to suppress Li dendrite growth [37]. It has been further demonstrated that both pressure‐driven and density‐driven mechanisms can stabilize electrodeposition in metal anodes [38]. Subsequently, electro‐chemo‐mechanical coupling was shown to govern Li deposition stability at Li–SE interfaces and to establish the competition between mechanical and electrical overpotentials in determining the interfacial reaction distribution [39]. More recently, an adapted chemo‐mechanical model demonstrated that the soft–hard pairing rule suppresses alloy dendrites; however, this model does not explicitly account for practical operating conditions such as stack pressure, temperature, and applied current density [40]. In order to make more accurate predictions for the stability of alloy‐SE interfaces in practical applications, it is crucial to develop electro‐chemo‐mechanical models that account for both homogeneous and heterogeneous alloy‐SE interfaces.

In this study, we consider both homogeneous and heterogeneous interface models to investigate the interface stability and the initiation of alloy dendrites in alloy‐SE systems. The critical stack pressure is defined as the model‐predicted minimum stack pressure required to maintain interfacial stability between the solid electrolyte and the alloy anode and is determined for both models. We find that the homogeneous interface model presents a higher critical stack pressure, making it a bottleneck. Based on thermodynamic and mechanical considerations, we propose three material design principles for low‐stack‐pressure SSBs: (1) utilize softer SEs with higher conductivity; (2) choose Li‐rich and harder alloy anode; and (3) control external conditions, such as low surface roughness amplitude (*A*), low applied current density (*I*_app_), and high operating temperature (*T*). High‐throughput screening of Li‐based binary alloys identifies Li–Al and Li–Sb as consistently stable anodes with SEs, highlighting their potential to enable low stack pressure operation due to mechanical stability in SSBs. Experiments show that the LiAl alloy paired with Li_6_PS_5_Cl (LPSC) exhibits stable cycling and smooth interface morphology when the stack pressure exceeds the critical stack pressure. In contrast, it shows poor performance and rough morphology below the critical stack pressure. This work provides material design guidelines to achieve low stack pressure and promote commercial applications.

## 2 Results and Discussion

### 2.1 Homogeneous Interface Model for Critical Stack Pressure Prediction

**FIGURE 1:**
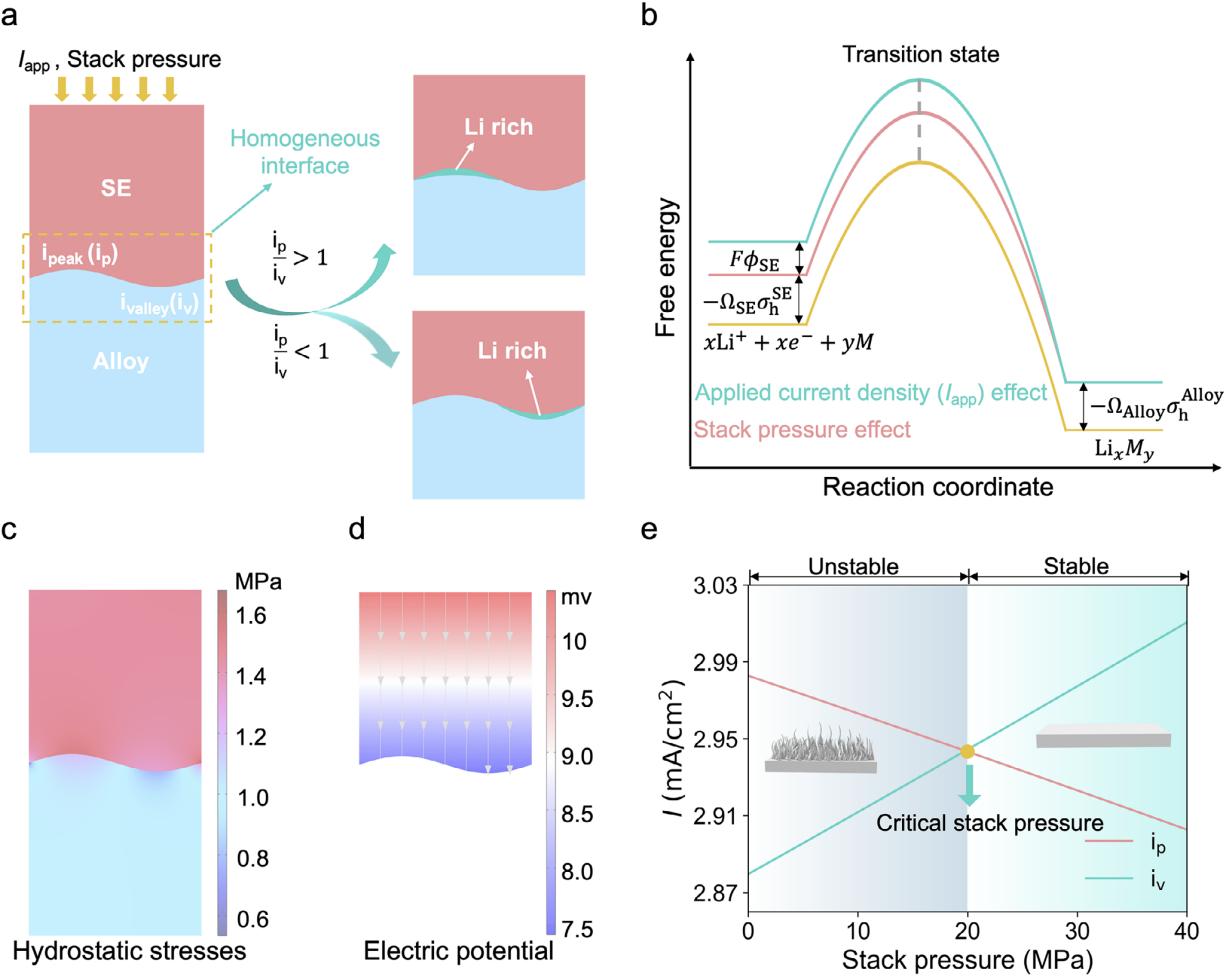
Homogeneous interface model for critical stack pressure predictions. (a) Geometry of an alloy−SE system with a homogeneous interface under *I*_app_ and stack pressure. Interface stability criterion: i_p_/i_v_ < 1 (stable), i_p_/i_v_ > 1 (unstable). (b) The free energy landscape of the redox reaction at the alloy‐SE interface in the homogeneous interface model is modulated by both *I*_app_ and stack pressure. (c–e) Hydrostatic stresses and electric potential distributions at 2 MPa stack pressure, 3 mA/cm^2^ current density, and room temperature (RT), for determining the critical stack pressure (i_p_ = i_v_) in LiAl–LPSC.

The schematic of the electro‐chemo‐mechanical coupling model for homogeneous alloy‐SE interfaces with roughness is presented in Figure 1a, referred as the homogeneous interface model. Details of the boundary conditions and parameters are provided in Tables S1 and S2. Under the stack pressure, alloys (at the bottom) and the SEs (at the top) initially form conformal contact. The interfacial electrochemical redox reaction during the charging process with *I*_app_ (lithiation) is described as

(1) $$xLi^{+}+xe^{-}+yM=Li_{x}M_{y}$$

where *M* denotes the alloying element with Li. This electrochemical reaction involves three key aspects: (1) the migration of Li^+^ ions through the SEs; (2) its subsequent reduction and alloying at the electrode‐electrolyte interface; and (3) the redistribution of current density (*I*) at the interface, which governs the reaction kinetics. The interface morphology is described by a sinusoidal function, *m*  =  *Asin*(ω*n*), where *A* and ω represent the surface roughness amplitude and frequency, respectively. The stability of the interface is quantified by the peak‐to‐valley current density ratio, defined as i_p_ divided by i_v_ (i_p_/i_v_). When i_p_/i_v_ > 1, the alloying reaction is biased, occurring faster at the peaks (y > 0) than valleys (y < 0), causing Li‐rich zones with greater volume expansion. In this case, morphological instability increases roughness, promoting Li alloy protrusions that grow into dendritic filaments, potentially penetrating the SE and causing short‐circuits. When i_p_/i_v_ < 1, the alloying reaction is more pronounced in the valley region, leading to a more uniform interface with less roughening. In this stable state, the interface remains smooth, reducing the likelihood of dendritic growth. When i_p_/i_v_ = 1, the initial interface morphology remains and shifts upward during charging.

Figure 1b illustrates the free energy profile of a redox reaction between Li^+^ and *M* at the alloy‐SE interface (yellow), with external *I*_app_ (cyan) and stack pressure (pink). The cyan curve illustrates the increase in free energy contributed by the *I*_app_, which originates from the electric potential of the SE ($\phi_{SE}$) and the Faraday constant (*F*) through the term *F* $\phi_{SE}$. Conversely, the pink curve shows the mechanical effect of the stack pressure via the term $-\Omega_{SE}\sigma_{h}^{SE}$. Owing to the high conductivity of the alloy, the variation in the electric potential of the alloy ($\phi_{Alloy}$) is negligible and can be approximated as zero. Consequently, the free energy of Li_*x*_ *M_y_* is influenced solely by the mechanical term $-\Omega_{Alloy}\sigma_{h}^{alloy}$. Here, Ω_*SE*_ represents the partial molar volume of Li^+^ in the SE, and Ω_*Alloy*_ denotes the partial molar volume of the alloy. Similarly, $\sigma_{h}^{alloy}$ and $\sigma_{h}^{SE}$ refer to the interfacial hydrostatic stresses in the alloy and the SE, respectively.

Section S1 provides the detailed derivation of critical stack pressure. As shown in Figure 1c, under conditions of 2 MPa stack pressure, 3 mA/cm^2^ current density, and RT, the LiAl and LPSC exhibit non‐uniform hydrostatic stress distributions. Notably, interfacial stresses are more compressive near peak compared to valley. Figure 1d further illustrates the spatially varying electric potential within the LPSC. Mechanical and electrical overpotentials, defined by Equations S5 and S6 respectively, develop non‐uniformly at the interface due to the observed stresses and electric potential variations.

Depending on the stack pressure relative to the critical stack pressure determined from Equation S11 (Figure 1e), there could be three distinct interface regimes. When stack pressure < critical stack pressure, the interface becomes morphologically unstable, developing surface roughening that promotes dendrite formation. Conversely, when the stack pressure is larger than the critical stack pressure, the interface roughness will be reduced, representing a dendrite‐free and stable interface. The critical threshold (the stack pressure equals to the critical stack pressure) represents a metastable state in which the interface maintains its initial morphology. This stack‐pressure‐dependent morphological evolution demonstrates how mechanical constraints can fundamentally alter interface stability in electrochemical systems.

Interfacial side reactions are inherently complex because they involve both chemical and electrochemical processes, and their products may vary with voltage [41]. Therefore, explicitly incorporating these reactions into the present model is challenging. Moreover, as shown in Figure S4, alloy anodes are less reactive than pure Li metal, suggesting a lower tendency for interfacial side reactions. It is true that plastic deformation and creep are important but are not specifically considered in this study. While alloy anodes typically undergo elastic‐to‐plastic deformation under stack pressure [30], this study focuses on the initial elastic regime within a simplified electro‐chemo‐mechanical framework. Consequently, the interfacial stability trend is thus approximated from the phase stability in the phase diagram during the initial linear‐elastic deformation stage. Under these assumptions, the predicted critical stack pressure represents an optimistic lower bound; an interface predicted as unstable under these simplified conditions would be even less stable under realistic operating conditions.

### 2.2 Spontaneous Homogenization in the Heterogeneous Interface Model

**FIGURE 2:**
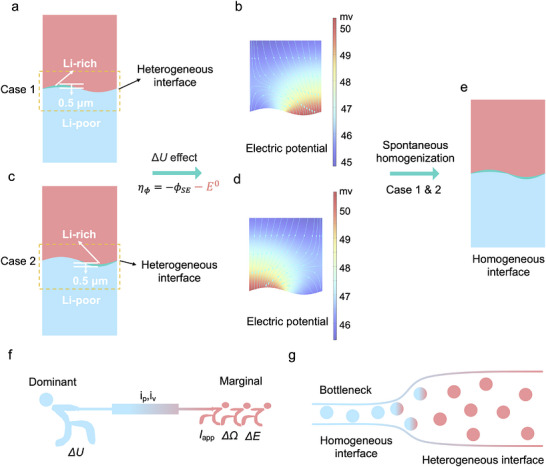
Heterogeneous interface model for critical stack pressure. (a,b) Case 1 exhibits Li‐rich phase (Li_3_Al_2_) at peaks and Li‐poor phase (LiAl) at valleys under 2 MPa stack pressure, 3 mA/cm^2^ current density, and RT. (c,d) Case 2 features Li_3_Al_2_ at valleys and LiAl at peaks under identical conditions. (e) The potential difference (Δ*U*) between LiAl and Li_3_Al_2_ drives heterogeneous‐to‐homogeneous interface evolution in both cases. (f) Schematic summary of multi‐factor competition regulating i_p_ and i_v_ in the heterogeneous interface model. (g) The homogeneous interface is the bottleneck compared to the heterogeneous interface in terms of critical stack pressure.

In a SSB system, during charging, the Li‐rich phase may tend to form either at the peak or valley region, leading to a heterogeneous alloy‐SE interface. Taking the LiAl–Li_3_Al_2_ system as an example, which corresponds to two adjacent phases in the phase diagram (Figure S11b). The preferential formation of the Li‐rich Li_3_Al_2_ phase within the LiAl anode could initially exhibit two distinct spatial distributions: (i) Case 1, where nucleation occurs predominantly at peak regions (Figure 2a), and (ii) Case 2, where phase segregation preferentially localizes in valley regions (Figure 2c). Figure 2b,d show that the electric potential streamlines consistently originate from Li_3_Al_2_ regions and terminate at LiAl regions across the interface. This directional streamline demonstrates a strong electrochemical driving force to transform LiAl zones into Li_3_Al_2_ zones. A similar trend of homogeneous lithiation is observed at other initial depths (0.25, 0.75, and 1 µm; Figures S1–S3), confirming that the potential difference (Δ*U*) between Li‐rich and Li‐poor phases consistently governs the interface evolution. The model parameters are detailed in Table S3.

Ultimately, the heterogeneous interface transitions into a homogeneous interface (Figure 2e). Figure S5 shows that stack pressure and *I*_app_ have only a marginal effect on the i_p_, i_v,_ and i_p_/i_v_ in the heterogeneous interface model. This could be attributed to the fact that the free energy landscape of the alloy‐SE interface redox reaction is primarily determined by the Δ*U* in the heterogeneous interface model, compared to stack pressure and *I*_app_. In other words, electro‐chemo‐mechanical coupling is insignificant for free energy in both cases of the heterogeneous interface model. The symbols Δ*E* and Δ*Ω* represent the differences in Young's modulus, and partial molar volume, respectively, between the Li‐poor and Li‐rich phases. Figures S6 and S7 confirm that Δ*U* dominates the interfacial stability in both cases, while Δ*E* and Δ*Ω* exhibit only marginal effects. Building upon the above analysis, Figure 2f provides a schematic summary of the multi‐factor competition governing the i_p_ and i_v_ in the heterogeneous interface model. Overall, the homogeneous interface presents greater critical stack pressure challenges compared to the heterogeneous interface (Figure 2g). Therefore, the homogeneous interface model, governing the critical stack pressure, has been further applied to establish the fundamental design principles for stable alloy‐SE interfacial cycling.

### 2.3 High κ_SE_ and Softer SEs

**FIGURE 3:**
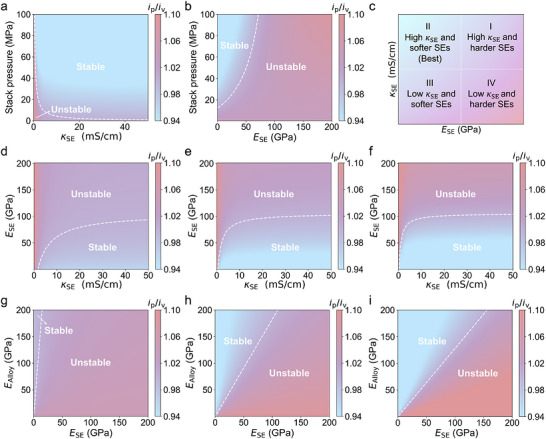
Stack‐pressure‐dependent interface stability descriptor (i_p_/i_v_) for the LiAl‐LPSC system at 3 mA/cm^2^ current density and RT, examined together with (a) ionic conductivity of SE (κ_SE_) and (b) Young's modulus of SE (*E*_SE_). (c) SE design principles based on κ_SE_ and *E*_SE_, with zone II as the optimal to achieve low stack pressure. (d–f) Relationship between κ_SE_ and *E*_SE_ under 15, 50, and 100 MPa stack pressure. (g–i) Mechanical matching stability between the SE and the Li alloy, characterized by the relationship between *E*_SE_ and Young's modulus of Li alloy (*E*_Alloy_) under 15, 50, and 100 MPa stack pressure.

The electrochemical performance of SSBs is influenced by the properties of SEs, including ionic conductivity (κ_SE_), chemical and electrochemical stability, interfacial compatibility and resistance [42, 43], and mechanical processability [44]. The influence of solid electrolyte (SE) characteristics on critical stack pressure has been systematically investigated, revealing their fundamental correlations. In Figure 3a, critical stack pressure (marked by the white dotted line for i_p_/i_v_ = 1) decreases significantly with increasing κ_SE_, indicating higher κ_SE_ is critical to maintain a small stack pressure. Consistent with this finding, recent studies [45, 46, 47] have strategically targeted κ_SE_ enhancement, demonstrating its validity as an effective approach for stack pressure minimization. Moreover, as shown in Figure 3b, the critical stack pressure predominantly increases as *E*_SE_ increase, suggesting that smaller *E*_SE_ contributes to interfacial stability. Smaller *E*_SE_ suggests reduced mechanical stiffness, indicating that the SE is relatively soft. The most favorable SE shall have high κ_SE_, softer SEs (region II in Figure 3c), while the least desirable ones locate in region IV with low κ_SE_ and higher *E*_SE_. Figure 3d–f illustrate that the stable region expands progressively with stack pressure increasing from 15, 50, to 100 MPa, further demonstrating that stack pressure enhances interface stability. When SEs pair with Li alloys, Figure 3g–i shows that stable regions emerge only when the *E*_SE_ is smaller than *E*_Alloy_, regardless of the stack pressure (15 MPa, 50 MPa, or 100 MPa). This indicates that the SE shall be mechanically softer than the Li alloy to ensure interface stability, which trend is more significant for small stack pressures. A stack pressure as low as 2 MPa also supports the conclusion above (Figure S8). Thus, the design principle for SE prioritizes high κ_SE_ and low *E*_SE_, smaller than *E*_Alloy_, to achieve low stack pressure, for stable SE‐anode interfaces during charging.

### 2.4 Li‐Rich and Harder Alloys

**FIGURE 4:**
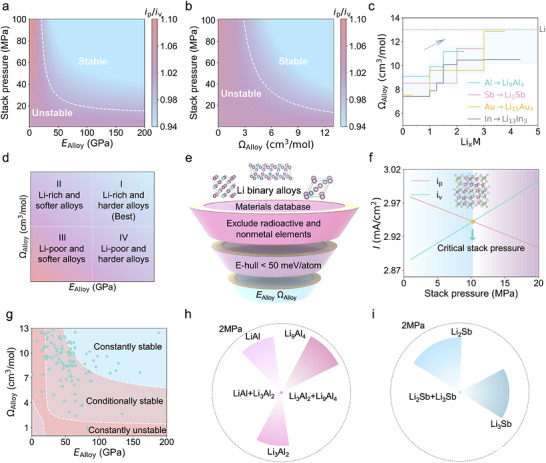
Design principles for Li alloys and high‐throughput screening for low critical stack pressure. For the LiAl‐LPSC under 3 mA/cm^2^ current density and RT, the i_p_/i_v_ as a function of stack pressure, combined with (a) *E*_Alloy_ and (b) Partial molar volume of Li alloy (Ω_Alloy_). (c) The relation between Ω_Alloy_ and Li concentration for various alloys. (d) Li alloy design principles based on *E*_Alloy_ and Ω_Alloy_. (e) High‐throughput computational workflow for screening Li binary alloys. (f) The minimum stack pressure required to stabilize the interface between the best Li binary alloys and LPSC. (g) Mechanics‐driven stability map for Li binary alloys paired with LSiGPSBO, based on critical stack pressure: constantly stable (<2 MPa), conditionally stable (2–100 MPa), and constantly unstable (>100 MPa). (h,i) Suggested Li alloy anodes with critical stack pressure < 2 MPa when paired with LSiGPSBO.

The mechanically relevant properties of alloy anodes, such as *E*_Alloy_ and Ω_Alloy_ (see Supporting Information for calculation details), are also closely related to the electrochemical performance. In Figure 4a, critical stack pressure decreases with increasing *E*_Alloy_, indicating modulus‐governed stabilization. Similarly, Figure 4b reveals an analogous trend for Ω_Alloy_, confirming mechanics‐dominated stabilization across both key parameters. To establish the relationship between Li concentration and Ω_Alloy_, we systematically analyzed four Li alloy compositions. Our results reveal an increase in Ω_Alloy_ with rising Li concentration (Figure 4c), suggesting that Li‐rich phases are statistically more likely to have higher Ω_Alloy_. Figure 4d presents a mechanics‐driven design principles of Li alloys using Ω_Alloy_ and *E*_Alloy_ as key descriptors. In region I, Li‐rich alloys (high Ω_Alloy_) with high stiffness (high *E*_Alloy_) are optimal for minimizing stack pressure. In contrast, regions II and IV are less favorable, while region III with Li‐poor and mechanically soft alloys is the least favorable.

To reduce the critical stack pressure, the optimal alloy shall be Li‐rich with high hardness, while the ideal SE should possess high κ_SE_ and be mechanically softer than the alloy. We now aim to evaluate whether currently available alloy and SE materials meet commercial low stack pressure requirements. To this end, a high‐throughput computational workflow has been employed to screen all Li binary alloys in the database. As shown in Figure 4e, the Materials Project database contains 276 Li‐containing binary alloys. After excluding radioactive/nonmetallic elements and removing duplicate entries, 148 candidates remain. Further screening based on e‐hull < 50 meV/atom yields 111 thermodynamically stable Li binary alloys. For these selected alloys, we calculated their *E*_Alloy_ and Ω_Alloy_. The upper bounds for *E*_Alloy_ and Ω_Alloy_ were set at 200 GPa and 13 cm^3^/mol, based on the values from LiPt_2_ and pure Li, respectively, to determine the minimum stack pressure. Our simulations (Figure 4f) reveal that the critical stack pressure is approximately 11 MPa, with interfaces becoming constantly unstable under 10 MPa (Figure S9a). As the stack pressure increases to 20 MPa, the stable region becomes larger (Figure S9b,c). Notably, 11 MPa exceeds the commercial benchmark of less than 2 MPa. Consequently, with LPSC serving as the SE for Li binary alloys, merely tuning the mechanical properties of the alloys is inadequate to satisfy the stack pressure requirements for industrial applications.

Given this limitation, based on the design principle to select other SEs that can intrinsically meet the low stack pressure demands. Among the reported typical SEs, we compare sulfide‐based, thin‐film, garnet‐type, NASICON‐type, halide‐based, and anti‐perovskite SEs. Based on the above SE design principles, SEs with high κ_SE_ and low *E*_SE_ perform the best, such as Li_9.54_[Si_0.6_Ge_0.4_]_1.74_P_1.44_S_11.1_Br_0.3_O_0.6_ (LSiGPSBO) [48], as detailed in Table S4 and shown in Figure S10. Figure 4g presents a mechanics‐driven stability regime map for Li binary alloys paired with LSiGPSBO under a current density of 3 mA/cm^2^ and RT. The interface stability is categorized into three regions based on the critical stack pressure. When critical stack pressure is less than 2 MPa, this case is classified as constantly stable (Table S5), meeting the practical requirements for commercial applications. When critical stack pressure falls between 2 and 100 MPa, this case is classified as conditionally stable, corresponding to the stack pressure range typically adopted in laboratory studies. When critical stack pressure exceeds 100 MPa, this case is classified as constantly unstable, as such high stack pressures are difficult to achieve in practical applications and thus have limited real‐world significance. Figure 4h,i reveal that the Li–Al and Li–Sb systems remain in the constantly stable region (critical stack pressure < 2 MPa) across all alloy phases in their phase diagrams, highlighting their potential as promising alloy anode systems from the perspective of mechanical stability in SSBs. Nevertheless, their practical applicability still faces additional challenges, including the synthesis of SEs with high κ_SE_ and low *E*_SE_, as well as alloy‐anode‐related issues such as electrochemical fatigue [49], diffusion limitations [50], and other degradation mechanisms [51].

### 2.5 Low *A*, Low *I*_app_, and High *T* External Conditions

**FIGURE 5:**
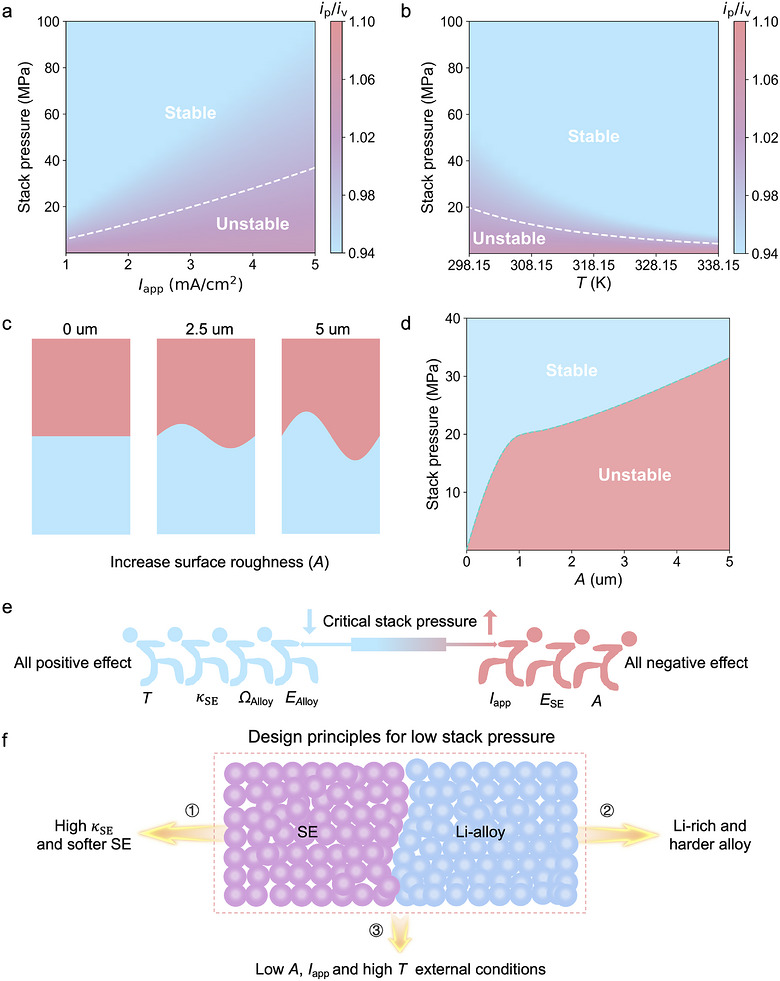
Design principles for external conditions. For the LiAl‐LPSC, the i_p_/i_v_ as a function of stack pressure, combined with (a) *I*_app_ at RT, and (b) *T* at 3 mA/cm^2^ current density. (c) Interface geometry of an alloy–SE system with a surface roughness amplitude (*A*) range of 0–5 µm. (d) Relationship between stack pressure and *A* in the LiAl‐LPSC system was examined under 3 mA/cm^2^ current density and RT. (e) Schematic summary of multi‐factor competition regulating critical stack pressure. (f) Overview of the key design principles governing Li alloy, SE, and external conditions for low stack pressure.

Building on previous studies that have made valuable contributions to low‐stack‐pressure operation in SSBs by focusing on electrolyte or electrode design [52, 53, 54], our work examines the combined effects of alloy anodes, SEs, and external conditions (i.e., *A*, *I*_app_, and *T*). As shown in Figure 5a, with an increase in *I*_app_ from 1 mA/cm^2^ to 5 mA/cm^2^, the required critical stack pressure also increases, which is consistent with the experimental observation that higher *I*_app_ leads to interface instability (dendrite formation) [28]. The decrease in critical stack pressure with increasing *T* (Figure 5b) suggests that elevated *T* stabilizes the interface morphology, in agreement with prior experimental findings [55]. Figure 5c illustrates how increasing *A* (from 0 µm to 5 µm) modifies the interfacial morphology between alloy and SE, transforming a flat interface into an increasingly corrugated one. A smaller value of *A* leads to reduced critical stack pressure (Figure 5d), however, achieving *A* = 0 µm is challenging and largely depends on the level of manufacturing technology. Figure 5e demonstrates the competitive regulation of critical stack pressure by thermodynamic and mechanical factors. While higher values of *T*, κ_SE_, *Ω*_Alloy_, and *E*_Alloy_ reduce the critical stack pressure (positive effects), increased *I*_app_, *E*_SE_, and *A* elevate it (negative effects). The interplay of these seven factors collectively determines critical stack pressure. As shown in Figure 5f, to achieve low critical stack pressure, three material design principles should be considered: (1) using softer, high‐conductivity SEs (softer than the Li‐alloy); (2) employing Li‐rich, harder alloy anodes; and (3) operating under low *I*_app_ and elevated *T*. These three design principles can be applied independently or simultaneously to reduce critical stack pressure.

### 2.6 Experimental Validation

**FIGURE 6:**
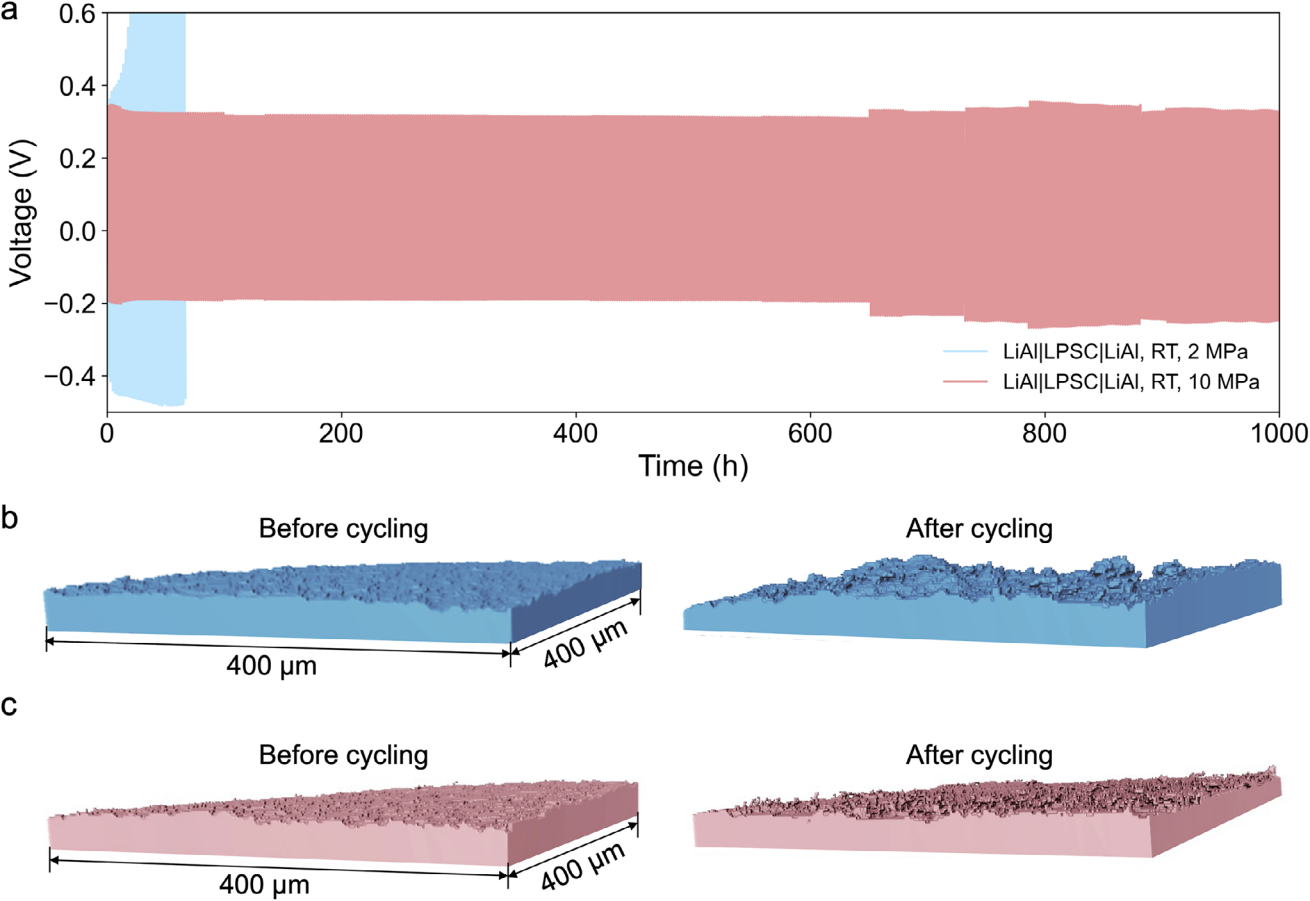
Electrochemical performance of Li‐Al alloy. (a) The electrochemical performance of LiAl|LPSCl|LiAl symmetric cells was evaluated by cycling at a current density of 1 mA/cm^2^ and an areal capacity of 1 mAh/cm^2^ under stack pressures of 2 MPa (below the critical stack pressure) and 10 MPa (above the critical stack pressure). Comparative 3D renderings of the subvolumes under (b) 2 MPa and (c) 10 MPa stack pressure, before and after symmetric cell cycling, obtained by x‐ray CT analysis.

To validate our electro‐chemo‐mechanical model and the effect of stack pressure on interfacial morphology, we synthesized LiAl foils (Figure S11a). Both SEM imaging and EDS mapping (surface and cross‐sectional views in Figure S12) demonstrate a uniform distribution of Al across the fabricated foils. Under RT and 1 mA/cm^2^ current density, simulation show that LiAl|LPSC|LiAl symmetric cells require a critical stack pressure of 6 MPa. Therefore, applied stack pressures of 2 MPa and 10 MPa represent conditions below and above the critical threshold, respectively. Cells cycled at 2 MPa exhibited significant voltage hysteresis, reaching up to 5 V within 100 h (Figure S13). In contrast, cells operated at 10 MPa demonstrated stable voltage profiles for over 1000 h (Figure 6a). When the stack pressure was further increased to 15 MPa (Figure S14), the cells exhibited similar reversibility and capacity retention as those under 10 MPa during initial cycling. However, from 300 to 500 cycles, larger voltage hysteresis was observed. This phenomenon suggests that maintaining an appropriate stack pressure is necessary, rather than simply applying excessively high stack pressures.

X‐ray computed tomography (XCT) was employed to characterize the morphological changes of the LiAl anode before and after cycling in LiAl|LPSC|LiAl symmetric cells. Representative 2D slice images obtained at different states are presented in Figure S15. Following 3D reconstruction, the morphological evolution of the LiAl alloy under applied pressures of 2 and 10 MPa was visualized, as shown in Figure 6b,c, respectively. A subvolume of the alloy with an edge length of 400 µm was extracted for detailed analysis. We quantified the surface roughness based on X‐ray CT data obtained from LiAl|LPSC|LiAl symmetric cells under stack pressures of 2 MPa and 10 MPa using Equation (2). *S_a_* represents the arithmetic mean surface roughness and is calculated as the average absolute deviation of the surface height (*Z_i_*) from the mean surface height ($\bar{Z}$) over all sampled points. Under a stack pressure of 2 MPa, the *S_a_* of the LiAl alloy increased from 1.22 µm before cycling to 4.11 µm after cycling, indicating significant surface roughening associated with dendritic growth that could potentially penetrate the LPSC. In contrast, under 10 MPa, the *S_a_* value increased only from 1.33 µm before cycling to 1.90 µm after cycling, suggesting a more uniform Li alloying process and more effective suppression of dendrite formation. Additional surface and cross‐sectional SEM observations (Figure S16) further confirmed the enhanced morphological stability of the LiAl alloy under 10 MPa stack pressure, showing a flatter surface after cycling compared to 2 MPa, which is consistent with the XCT findings.

(2) $$S_{a}=\frac{1}{N}\sum_{i=1}^{N}\left|Z_{i}-\bar{Z}\right|$$

An additional Li_17_Sn_4_/LPSCl experiment was conducted to further validate the electro‐chemo‐mechanical model predictions. Simulations indicate that under RT and a current density of 1 mA/cm^2^, Li_17_Sn_4_|LPSC|Li_17_Sn_4_ symmetric cells require a critical stack pressure of 9 MPa. Therefore, applying stack pressures of 5 MPa and 15 MPa corresponds to operating below and above this critical threshold, respectively. As illustrated in Figure S18, cells cycled at 5 MPa experienced pronounced voltage hysteresis, reaching up to 2 V within 150 h. In contrast, cells subjected to 15 MPa maintained stable voltage behavior for more than 500 h. We also reviewed published studies and observed consistent trends in systems such as Li‐In, Li‐Sn, Li‐Ag, Li‐Mg, Li‐Zn, and Na‐Sn, as shown in Figure S20 [11, 40, 56, 57, 58, 59, 60]. In symmetric cells where dendrites form and the cells become unstable, the i_p_/i_v_ is greater than 1. In contrast, in stable symmetric cell cycling, the i_p_/i_v_ remains below 1, regardless of whether the experiments are conducted at RT or elevated temperatures. These findings, with detailed information summarized in Tables S7–S9, support our simulation results and demonstrate the universality of our model across various alloy systems, further suggesting its applicability to Na‐based alloys as well.

## 3 Conclusion

In summary, this work develops an electro‐chemo‐mechanical modeling framework combined with X‐ray CT characterization to predict the minimum stack pressure required to maintain interfacial stability between alloy anodes and solid electrolytes, termed the critical stack pressure. Three material design principles were established to enable low stack pressure operation: (1) utilizing high κ_SE_ and softer SEs; (2) using Li‐rich and harder alloy anodes; and (3) operating under conditions of elevated *T* and reduced *I*_app_. Subsequently, we developed a high‐throughput computational workflow to screen all Li binary alloys within the database, which led to the identification of the Li–Al and Li–Sb systems as the most promising candidates for low stack pressure operation, as they remained in the constant stable region (<2 MPa). Ultimately, experimental validation of the Li–Al system confirmed that applying a stack pressure slightly above the critical stack pressure leads to a more stable interface morphology and better cycling performance, thereby supporting our simulation. Our results suggest that progress toward practical application may be promoted by optimizing the SE, alloy anode, and external conditions according to these principles, while applying an appropriate stack pressure rather than simply increasing it as much as possible.

## Author Contributions

**Yuping Huang**: conceptualization, methodology, data curation, writing – original draft. **Xinyu Yu**: data curation. **Hong Zhu**: supervision, resources, writing – review and editing, funding acquisition. **Chen Su**: investigation. **Jia Li**: investigation. **Shou‐Hang Bo**: writing – review and editing, supervision, resources, funding acquisition. **Zhe‐Tao Sun**: methodology. **Shiwei Chen**: methodology.

## Conflicts of Interest

The authors declare no conflicts of interest.

## Supporting information




**Supporting File**: advs76946‐sup‐0001‐SuppMat.docx.

## Data Availability

The data that support the findings of this study are available from the corresponding author upon reasonable request.
