# Supplementary material for: Design Principles for Alloy‐Anode‐Based Low‐Stack‐Pressure Solid‐State Batteries
Source: Adv Sci (Weinh). 2026 Aug 3;13(60):e76946. doi: 10.1002/advs.76946 (PMC13430935; doi:10.1002/advs.76946)
Supplement: Supplementary file 1 [file ADVS-13-e76946-s001.docx]

**Supporting Information**

**Design Principles for Alloy-Anode-Based Low-Stack-Pressure Solid-State Batteries**

Yuping Huang ^a,b^, Zhe-Tao Sun ^a,b^, Xinyu Yu ^a,b^, Shiwei Chen ^a,b^, Chen Su^c^, Jia Li ^a,b^,

Shou-Hang Bo ^b,c,^*, Hong Zhu^a,^*

^a^ Global College, Shanghai Jiao Tong University, 800, Dongchuan Road, Shanghai 200240, China

^b^ Future Battery Research Center, Global Institute of Future Technology, Shanghai Jiao Tong University, Shanghai 200240, China

^c^ School of Chemistry and Chemical Engineering, Shanghai Jiao Tong University, Shanghai 200240, China

Corresponding Author *E-mail: shouhang.bo@sjtu.edu.cn, hong.zhu@sjtu.edu.cn.

**Methods**

**First-principles** **calculations**

All DFT calculations were carried out using the Vienna Ab initio Simulation Package (VASP) with the projector augmented wave (PAW) method.^[1]^ The Perdew-Burke-Ernzerhof (PBE) generalized-gradient approximation (GGA)^[2]^ was employed, with an energy cutoff set to 520 eV. Additionally, the k-point density was chosen to align with the standards used in the Materials Project database. The energy and force convergence thresholds were set to 10^-5^ eV and 0.01 eV/Å, respectively.

**Partial molar volume** **calculations**

We utilized the Bader charge^[3]^ approach to calculate the static Li^+^ volume within the electrolyte, where the Bader charge on an atom corresponds to the total charge enclosed within its atomic volume (Bader volume). For the partial molar volume of the alloy, consider LiAl (Al/LiAl) as an example. The phase undergoes a transition from Al to LiAl as the Li content increases by Δn_Li_. In this case, the partial molar volume can be expressed as Ω_LiAl_ = (V_LiAl_ – V_Al_) / Δn_Li_.^[4]^

**Cell assembly**

The LiAl|LPSC|LiAl and Li_17_Sn_4_|LPSC|Li_17_Sn_4_ symmetric cells were fabricated using a similar procedure. First, 100 mg of Li_6_PS_5_Cl (LPSC) powder was cold-pressed at 50 MPa for 1 min to form the solid electrolyte pellet. Aluminum foil (40 μm thickness, 10 mm diameter) or tin foil (20 μm thickness, 10 mm diameter) was then bonded to both sides of the pellet under 370 MPa pressure for 10 min. Subsequently, lithium foil was pressed onto the Al or Sn layers at Li:Al ≈ 1:1 and Li:Sn ≈ 17:4 molar ratios, respectively, under 100 MPa pressure, followed by a 6 h resting period to facilitate the spontaneous formation of LiAl and Li_17_Sn_4_ alloys. All cell assembly procedures were conducted in an argon-filled glove box with oxygen and moisture levels maintained below 0.01 ppm.

**X-ray computed tomography experiments**

X-ray computed tomography (XCT) scans were acquired on an Xradia 520 Versa instrument, capable of achieving a spatial resolution of 700 nm. To minimize air exposure, all samples were wrapped with Kapton tape prior to being secured in the XCT sample stage for three-dimensional analysis of the alloy anode morphology. Instrument parameters included an X-ray source voltage of 80 kV and a detector pixel size of 1.6 μm.

The electro-chemo-mechanical model includes two domains: alloy and SE. Equation S1 describes the transport of Li^+^ ions in the SE, governed by the charge conservation equation, with concentration gradients being negligible due to the single-ion conduction property of the SE. Equation S2 describes electron transport in Li alloy, governed by the charge conservation equation, with the variation in electric potential considered negligible due to the high conductivity of alloy anode.

$$\begin{aligned} \nabla\cdot\left( \kappa_{\text{SE}}\nabla\phi_{SE} \right)=0\#\left( 1 \right) \end{aligned}$$

$$\begin{aligned} \nabla\cdot\left( \sigma_{e}\nabla\phi_{Alloy} \right)=0\#\left( 2 \right) \end{aligned}$$

**Section S1: Homogeneous/heterogeneous interface model formula derivation**

$$\begin{aligned} x\mathrm{Li}^{+}+{xe}^{-}+yM=\mathrm{Li}_{x}M_{y}\#\left( 3 \right) \end{aligned}$$

$$\begin{aligned} U=\frac{-\Delta G}{F}\#\left( 4 \right) \end{aligned}$$

Where *U* is the equilibrium potential of the alloying reaction under standard temperature and pressure, and $\Delta G$represents the Gibbs free energy change of the alloying reaction.

$$\begin{aligned} \eta_{\sigma}=\frac{\Omega_{SE}\sigma_{h}^{SE}-\Omega_{Alloy}\sigma_{h}^{Alloy}}{F}\#\left( 5 \right) \end{aligned}$$

$\eta_{\sigma}$ is mechanical overpotential, $\Omega_{Alloy}$denotes the partial molar volume of alloy, $\Omega_{SE}$represents the partial molar volume of Li^+^ in the solid-state electrolyte (SE), while $\sigma_{h}^{alloy}$ and $\sigma_{h}^{SE}$ refer to the interfacial hydrostatic stresses in alloy and the SE, respectively.

$$\begin{aligned} \eta_{\phi}=\phi_{Alloy}-\phi_{SE}-U=-\phi_{SE}-U\#\left( 6 \right) \end{aligned}$$

$\eta_{\phi}$ is electrical overpotential, and $\phi_{SE}$ is the electric potential of SE, $\phi_{Alloy}$is the electric potential of alloy. The variation in $\phi_{Alloy}$is negligible and therefore considered to be zero. In the homogeneous interface model, the *U* is set to 0 V as a reference. Consequently, the Δ*U* between peak and valley of the alloy remains zero. In contrast, for the heterogeneous interface model, *U* adopts the potential of the alloy phases, resulting in non-zero Δ*U* values between peaks and valleys. This Δ*U* actively drives the transition from heterogeneous to homogeneous interfaces.

$$\begin{aligned} V=U-\eta_{\sigma}\#\left( 7 \right) \end{aligned}$$

$V$ represents the equilibrium potential with mechanical overpotentials.

$$\begin{aligned} \eta=\phi_{Alloy}-\phi_{SE}-V=\phi_{Alloy}-\phi_{SE}-U+\eta_{\sigma}=\eta_{\phi}+\eta_{\sigma}\#\left( 8 \right) \end{aligned}$$

𝜂 is total overpotential, $i$ is the net current of the systems.

$$\begin{aligned} i= i_{0}\left[ \exp\left( \frac{\left( 1-\alpha\right)F\eta}{RT} \right)-\exp\left( -\frac{\alpha F\eta}{RT} \right) \right] \#\left( 9 \right) \end{aligned}$$

$$\begin{aligned} i_{0}=i_{00}\exp\left( \frac{F\eta_{\sigma}}{2RT} \right) \#\left( 10 \right) \end{aligned}$$

The current density with the mechanics factor is denoted as $i_{0}$, while $i_{00}$ represents the exchange current density independent of mechanics. Additionally, F stands for the Faraday constant, R for the universal gas constant, and T for the temperature. It is commonly observed that the electric overpotential makes an equal contribution to both the forward and backward halves ($\alpha$=0.5), whereas the mechanical overpotential selectively contributes to the forward half.

Referring to equations 8 to 10, we can derive the expression for $i_{\sigma-\phi}$.

$$\begin{aligned} {i_{\sigma-\phi}= i}_{00}\left[ \exp\left( \frac{2F\eta_{\sigma}+F\eta_{\phi}}{2RT} \right)-\exp\left( -\frac{F\eta_{\phi}}{2RT} \right) \right]\#\left( 11 \right) \end{aligned}$$

$i_{\sigma-\phi}$is the exchange current that takes into account both mechanical and electric potential effect. Based on i_p_ = i_v_, we can determine the critical stack pressure. The high temperature effect is given by the following equation:

$$\begin{aligned} \kappa=\kappa_{25℃}\exp\left( -\frac{E_{a}}{R}\left( \frac{1}{T}-\frac{1}{T_{25℃}} \right) \right)\#\left( 12 \right) \end{aligned}$$

$$\begin{aligned} i_{00}=i_{00,25℃}\exp\left( -\frac{E_{i_{00}}}{R}\left( \frac{1}{T}-\frac{1}{T_{25℃}} \right) \right)\#\left( 13 \right) \end{aligned}$$

$\kappa$ is ionic conductivity (S/m), T is the operational temperature of the cell. $E_{a}$ and $E_{i_{00}}$ represent the activation energies of SE and charge transfer, respectively. $\kappa_{25℃}$and $i_{00,25℃}$ denote the SE ionic conductivity and the exchange current densityat 25°C.

Table S1. Boundary conditions for the Alloy-SE domain under stack pressure and current density.

| Domain | Boundary | Mechanics  $\overset{⃑}{\nabla}\cdot\overset{⃡}{\sigma}=0$ | Li^+^/Na^+^ ion transport $\nabla\cdot\left( \kappa_{\text{SE}}\nabla\phi_{SE} \right)=0$ |
| --- | --- | --- | --- |
| SE | Top surface ($y=H_{SE}$) | $\overset{⃡}{\sigma}\vec{n}=P_{stack}\vec{n}$ | $-\kappa_{\text{SE}}\nabla\phi_{SE}= I$ |
|  | Alloy-SE interface  ($y=Asin(\omega x)$) | / | $-\kappa_{\text{SE}}\nabla\phi_{SE}= i_{\sigma-\phi}$ |
|  | Lateral faces  ($x=0,W$) | $u_{x}=0, \frac{\partial u_{y}}{\partial x}=0$ | $-\kappa_{\text{SE}}\nabla\phi_{SE}=0$ |
| Alloy | Bottom surface  ($y=H_{Alloy}$) | $u_{x}=0,u_{y}=0$ | / |
|  | Lateral faces  ($x=0,W$) | $u_{x}=0, \frac{\partial u_{y}}{\partial x}=0$ | / |

Table S2. List of parameters used in the homogenous interface model for LiAl-LPSC.

| Parameters | Descriptions | Values | Units | Ref. |
| --- | --- | --- | --- | --- |
| $E_{Alloy}$ | Young’s modulus of alloy | 91.44 | GPa | This work |
| $\nu_{Alloy}$ | Poisson’s ratio of alloy | 0.19 | / | This work |
| $\Omega_{Alloy}$ | Partial molar volume of alloy | 9.11 | cm^3^/mol | This work |
| $E_{SE}$ | Young’s modulus of SE | 16.52 | GPa | This work |
| $\nu_{SE}$ | Poisson’s ratio of SE | 0.37 | - | This work |
| $\Omega_{SE}$ | Partial molar volume of SE | 2.66 | cm^3^/mol | This work |
| $\kappa_{25℃}$ | Effective ionic conductivity of SE at 298.15K | 0.21 | S/m | ^[5]^ |
| $\sigma_{e}$ | Electronic conductivity of Alloy | 1.1×10^7^ | S/m | ^[6]^ |
| $i_{00,25℃}$ | Mechanics-independent exchange current density | 10 | mA/cm^2^ | ^[7]^ |
| $E_{a}$ | Activation energy for ion transport in SE | 28.95 | kJ/mol | ^[8]^ |
| $E_{i_{00}}$ | Activation energy for charge transfer | 48.54 | kJ/mol | ^[9]^ |
| F | Faraday’s constant | 96485.33 | C/mol | / |
| R | Universal gas constant | 8.314 | J/mol/K | / |
| T | Operating temperature | Specified | K | / |
| $W$ | Domain width | 20 | $um$ | / |
| $H_{Alloy}$ | Thickness of Alloy | 20 | $um$ | / |
| $H_{SE}$ | Thickness of SE | 20 | $um$ | / |
| $A$ | Surface roughness amplitude | 1 | $um$ | ^[6]^ |
| $\omega$ | Surface roughness frequency | $2\pi/W$ | $m^{-1}$ | ^[10]^ |

Table S3. List the parameters used in the heterogeneous interface model for Li_3_Al_2_ and LiAl as anodes, with LPSC as SE.

| Parameters | Descriptions | Values | Units | Ref. |
| --- | --- | --- | --- | --- |
| $E_{\mathrm{LiAl}}$ | Young’s modulus of alloy | 91.44 | GPa | This work |
| $\nu_{\mathrm{LiAl}}$ | Poisson’s ratio of alloy | 0.19 | / | This work |
| $\Omega_{\mathrm{LiAl}}$ | Partial molar volume of alloy | 9.11 | cm^3^/mol | This work |
| $U_{\mathrm{LiAl}}$ | Potential of alloy | 0.20 | V | This work |
| $E_{\mathrm{Li}_{3}\mathrm{Al}_{2}}$ | Young’s modulus of alloy | 81.06 | GPa | This work |
| $\nu_{\mathrm{Li}_{3}\mathrm{Al}_{2}}$ | Poisson’s ratio of alloy | 0.15 | / | This work |
| $\Omega_{\mathrm{Li}_{3}\mathrm{Al}_{2}}$ | Partial molar volume of alloy | 9.92 | cm^3^/mol | This work |
| $U_{\mathrm{Li}_{3}\mathrm{Al}_{2}}$ | Potential of alloy | 0.05 | V | This work |
| $E_{SE}$ | Young’s modulus of SE | 27.20 | GPa | This work |
| $\nu_{SE}$ | Poisson’s ratio of SE | 0.34 | / | This work |
| $\Omega_{SE}$ | Partial molar volume of SE | 2.66 | cm^3^/mol | This work |
| $\kappa_{25℃}$ | Effective ionic conductivity of SE at 298.15K | 0.3 | S/m | ^[5]^ |
| $\sigma_{e}$ | Electronic conductivity of Alloy | 1.1×10^7^ | S/m | ^[6]^ |
| $i_{00,25℃}$ | Mechanics-independent exchange current density | 10 | mA/cm^2^ | ^[7]^ |
| $E_{a}$ | Activation energy for ion transport in SE | 28.95 | kJ/mol | ^[8]^ |
| $E_{i_{00}}$ | Activation energy for charge transfer | 48.54 | kJ/mol | ^[9]^ |
| F | Faraday’s constant | 96485.33 | C/mol | / |
| R | Universal gas constant | 8.314 | J/mol/K | / |
| T | Operating temperature | Specified | K | / |
| $W$ | Domain width | 20 | $um$ | / |
| $H_{Alloy}$ | Thickness of Alloy | 20 | $um$ | / |
| $H_{SE}$ | Thickness of SE | 20 | $um$ | / |
| $A$ | Surface roughness amplitude | 1 | $um$ | ^[6]^ |
| $\omega$ | Surface roughness frequency | $2\pi/W$ | $m^{-1}$ | ^[10]^ |

Figure S1. Electric potential distribution in the heterogeneous interface model at a lithiation depth of 0.25 μm under 2 MPa stack pressure, 3 mA/cm^2^ current density, and room temperature (RT). (a, b) LiAl at the valley (blue) and Li_3_Al_2_ at the peak (green). (c, d) LiAl at the peak (blue) and Li_3_Al_2_ at the valley (green).

Figure S2. Electric potential distribution in the heterogeneous interface model at a lithiation depth of 0.75 μm under 2 MPa stack pressure, 3 mA/cm^2^ current density, and RT. (a, b) LiAl at the valley (blue) and Li_3_Al_2_ at the peak (green). (c, d) LiAl at the peak (blue) and Li_3_Al_2_ at the valley (green).

Figure S3. Electric potential distribution in the heterogeneous interface model at a lithiation depth of 1 μm under 2 MPa stack pressure, 3 mA/cm^2^ current density, and RT. (a, b) LiAl at the valley (blue) and Li_3_Al_2_ at the peak (green). (c, d) LiAl at the peak (blue) and Li_3_Al_2_ at the valley (green).

Figure S4. Reaction energies between alloy anodes and solid-state electrolytes. (a) Li–Al alloys; (b) Li–Sn alloys.

Figure S5. Heterogeneous interface model. The distribution of hydrostatic stresses and the variations in peak current (i_p_) and valley current (i_v_) with increasing stack pressure (*P*_stack_) or applied current density (*I*_app_). (a~c) LiAl at the valley and Li_3_Al_2_ at the peak under 2 MPa stack pressure, 3 mA/cm^2^ current density, and RT. (d~f) LiAl at the peak and Li_3_Al_2_ at the valley under 2 MPa stack pressure, 3 mA/cm^2^ current density, and RT.

Figure S6. Multiscale determinants of heterogeneous interface stability (Li_3_Al_2_ at peak, LiAl at valley): Δ*U* dominant effect, others are marginal effects under 2MPa stack pressure, 3mA/cm^2^ current density, and RT. (a) Potential difference between LiAl and Li_3_Al_2_ (Δ*U*), (b) Partial molar volume difference (Δ*Ω*), (c) Young's modulus difference (Δ*E*).

Figure S7. Multiscale determinants of heterogeneous interface stability (Li_3_Al_2_ at valley, LiAl at peak): Δ*U* dominant effect, others are marginal effects under 2MPa stack pressure, 3mA/cm^2^ current density, and RT. (a) Δ*U*, (b) Δ*Ω*, (c) Δ*E*.

Figure S8. At 2 MPa stack pressure, 3 mA/cm^2^ current density, and RT, the interfacial stability (iₚ/iᵥ) of the LiAl–LPSC system is shown as a function of the *E*_SE_, with (a) varying $\kappa_{\text{SE}}$ and (b) varying *E*_Alloy_.

Figure S9. Relationship between the size of the stable region and stack pressure at (a) 10 MPa, (b) 11 MPa and (c) 20 MPa.

Figure S10. Compare various SEs based on their Young’s modulus and conductivity.

Figure S11. (a) X-ray diffraction patterns (XRD) for LiAl. (b) phase diagram of the Li-Al alloy.

The XRD patterns of the as-synthesized alloys are presented in Figure S11a. The formation of composite phases, including Al and LiAl, is observed in the LiAl alloy, which is consistent with the corresponding phase diagrams (Figure S11b). All reference peaks were obtained from the ICSD database (Al: #18839; LiAl: #57950).

Figure S12. Surface and cross-sectional SEM images along with EDS elemental mapping of the LiAl alloy.

Figure S13. LiAl|LPSC|LiAl symmetric cells cycled at 1 mA/cm^2^ current density and 1 mAh/cm^2^ areal capacity under 2 MPa stack pressure.

Figure S14. LiAl|LPSC|LiAl symmetric cells cycled at 1 mA/cm^2^ current density and 1 mAh/cm^2^ areal capacity under 15 MPa stack pressure.

Figure S15. Reconstructed 2D image slices before and after cycling of the LiAl|LPSC|LiAl symmetric cells under stack pressure of (a) 2 MPa and (b) 10 MPa.

Figure S16. Surface and cross-sectional SEM images of symmetric cells after cycling under stack pressures of (a,b) 2 MPa and (c,d) 10 MPa.

Figure S17. (a) X-ray diffraction patterns (XRD) for Li_17_Sn_4_ alloy anode. (b) Surface and cross-sectional SEM images along with EDS elemental mapping of the Li_17_Sn_4_ alloy anode.

The XRD patterns of the as-synthesized Li_17_Sn_4_ alloys are presented in Figure S17a. Li_17_Sn_4_ appears to be primarily composed of Li_17_Sn_4_. All reference peaks were obtained from the ICSD database (Li_17_Sn_4_: #107215). Both SEM imaging and EDS mapping of the surface and cross-sectional views in Figure S17b demonstrate a uniform distribution of Sn across the fabricated foils.

Figure S18. The electrochemical performance of Li_17_Sn_4_|LPSC|Li_17_Sn_4_ symmetric cells was evaluated by cycling at a current density of 1 mA/cm^2^ and an areal capacity of 1 mAh/cm^2^ under stack pressures of 5 MPa (below the critical stack pressure) and 15 MPa (above the critical stack pressure).

Figure S19. Nyquist plots of Li_17_Sn_4_|LPSC|Li_17_Sn_4_ symmetric cells before and after cycling under different stack pressures. (a) 5MPa (below the critical stack pressure), (b) 15MPa (above the critical stack pressure). The equivalent circuit used for fitting is shown in the inset, where R_1_ corresponds to the bulk resistance and R_2_ represents the interfacial resistance.

Figure S19 illustrates the typical EIS responses of Li_17_Sn_4_|LPSC|Li_17_Sn_4_ symmetric cells before and after cycling under 5 and 15 MPa pressures. Combined with the fitting parameters in Table S6, it can be seen that the post-cycling R_2_ under 15 MPa is much lower than that under 5 MPa, suggesting that operating above the critical stack pressure effectively mitigates interfacial resistance growth and maintains better interfacial stability.

Figure S20. Comparison between experimental results and our model predictions across various Li/Na alloys.

Table S4. Detail information of candidate SEs.

| **SEs** | $\boldsymbol{\kappa}_{\text{SE}}$ **(mS/cm)** | ***E*_SE_ (GPa)** | **ref** |
| --- | --- | --- | --- |
| Li_9.54_[Si_0.6_Ge_0.4_]_1.74_P_1.44_S_11.1_Br_0.3_O_0.6_ | 32 | 20.972 | ^[11]^ |
| LiTaOCl_4_ | 12.4 | 19.995 | ^[12]^ |
| Li_7_La_3_Zr_2_O_12_ | 0.1~1 | 175.1 | ^[13]^ |
| Li_1.3_Ti_1.7_Al_0.3_(PO_4_)_3_ | 0.1~1 | 143.7 | ^[14]^ |
| Li_3_InCl_6_ | 1.03 | 38 | ^[15]^ |
| Li_3_BrSO_4_ | 10.9 | 42.180 | ^[16]^ |
| Li_3_OCl | 0.021 | 99.7 | ^[17]^ |
| LiPON | 2 × 10^−3^ | 77.0 | ^[18]^ |
| Li_6_PS_5_Cl | 2.1 | 16.52 | ^[19]^ |
| Li_10_GeP_2_S_12_ | 3.27 | 18.425 | ^[20]^ |

Table S5. Alloy phases in the constant stable region of Figure 4.

| Alloys | Phases |
| --- | --- |
| Li alloys | LiPt_2_, LiIr, LiPd_7_, LiRh, LiPd_3_, LiAl_3_, LiAl, LiB, Li_5_Ga_4_, Li_3_Al_2_,  Li_3_Ga_2_, LiPd, Li_2_Pt, Li_3_Ga_14_, Li_17_Sn_4_, Li_17_Pb_4_, Li_5_Sn_2_, Li_2_Si, Li_9_Al_4_, Li_2_Ga, Li_2_Al, Li_13_Si_4_, LiMg, LiAg_3_, Li_2_Sb, Li_3_Sb, Li_7_Ge_2_ |

Table S6. The fitting results of EIS for Li_17_Sn_4_.

| Phases | R_1_(Ω) | R_2_(Ω) |
| --- | --- | --- |
| 5MPa_before cycling | 42.5 | 3.6 |
| 5MPa_after cycling | 55.8 | 265.6 |
| 15MPa_before cycling | 29.6 | 2.1 |
| 15MPa_after cycling | 36.1 | 130.4 |

Table S7. Detail information of alloys.

| **Alloys** | $\boldsymbol{\Omega}_{\mathbf{Alloy}}$ **(cm^3^/mol)** | ***E*_Alloy_ (GPa)** |
| --- | --- | --- |
| Li_13_In_3_ | 12.21 | 42.50 |
| Li_17_Sn_4_ | 9.36 | 49.50 |
| Li_0.97_Mg_0.03_ | 12.09 | 6.86 |
| LiZn_2_ | 11.83 | 12.15 |
| Li_4_Ag | 10.33 | 17.43 |
| Li_3_Sn | 8.47 | 47.20 |
| Na_9_Sn_4_ | 16.74 | 24.97 |
| Na_10_Sn_4_ | 14.32 | 23.32 |
| Na_15_Sn_4_ | 19.10 | 20.17 |

Table S8. List of parameters used in the homogenous interface model for Na_15_Sn_4_-Na_3_SbS_4_.

| Parameters | Descriptions | Values | Units | Ref. |
| --- | --- | --- | --- | --- |
| $E_{Alloy}$ | Young’s modulus of alloy | 20.17 | GPa | This work |
| $\nu_{Alloy}$ | Poisson’s ratio of alloy | 0.28 | / | This work |
| $\Omega_{Alloy}$ | Partial molar volume of alloy | 19.10 | cm^3^/mol | This work |
| $E_{SE}$ | Young’s modulus of SE | 7.32 | GPa | This work |
| $\nu_{SE}$ | Poisson’s ratio of SE | 0.44 | - | This work |
| $\Omega_{SE}$ | Partial molar volume of SE | 6.48 | cm^3^/mol | This work |
| $\kappa_{25℃}$ | Effective ionic conductivity of SE at 298.15K | 0.025 | S/m | ^[21]^ |
| $\sigma_{e}$ | Electronic conductivity of Alloy | 1.1×10^7^ | S/m | ^[6]^ |
| $i_{00,25℃}$ | Mechanics-independent exchange current density | 4 | mA/cm^2^ | ^[22]^ |
| $E_{a}$ | Activation energy for ion transport in SE | 20.26 | kJ/mol | ^[21]^ |
| $E_{i_{00}}$ | Activation energy for charge transfer | 45.00 | kJ/mol | ^[23]^ |
| F | Faraday’s constant | 96485.33 | C/mol | / |
| R | Universal gas constant | 8.314 | J/mol/K | / |
| T | Operating temperature | Specified | K | / |
| $W$ | Domain width | 20 | $um$ | / |
| $H_{Alloy}$ | Thickness of Alloy | 20 | $um$ | / |
| $H_{SE}$ | Thickness of SE | 20 | $um$ | / |
| $A$ | Surface roughness amplitude | 1 | $um$ | ^[6]^ |
| $\omega$ | Surface roughness frequency | $2\pi/W$ | $m^{-1}$ | ^[6]^ |

Table S9. Validation of computational results against experimental data.

| Phase | Experiment | Computation | Consistency | Ref |
| --- | --- | --- | --- | --- |
| Li_13_In_3_/LPSC/ Li_13_In_3_ | Stable  (RT,1mA/cm^2^,45MPa,  1 mAh/cm^2^, >475 cls) | Stable  (i_p_/i_v_=0.824) | OK | ^[24]^ |
| Li_17_Sn_4_/LPSC/ Li_17_Sn_4_ | Stable  (RT,1mA/cm^2^,45MPa,  1 mAh/cm^2^, >475 cls) | Stable  (i_p_/i_v_=0.859) | OK | ^[24]^ |
| Li_0.97_Mg_0.03_/LPSC/ Li_0.97_Mg_0.03_ | Unstable  (RT,0.7mA/cm^2^,7MPa,  short-circuited) | Unstable  (i_p_/i_v_=1.053) | OK | ^[25]^ |
| Li_0.97_Mg_0.03_/LPSC/ Li_0.97_Mg_0.03_ | Unstable  (60°C,4mA/cm^2^,15MPa,  short-circuited) | Unstable  (i_p_/i_v_=1.253) | OK | ^[25]^ |
| LiZn_2_/LPSC/ LiZn_2_ | Unstable  (RT,2mA/cm^2^,70MPa,  short-circuited) | Unstable  (i_p_/i_v_=1.062) | OK | ^[26]^ |
| Li_4_Ag/LPSC/ Li_4_Ag | Stable  (RT,0.2mA/cm^2^,100MPa,  0.2 mAh/cm^2^, >75 cls) | Stable  (i_p_/i_v_=0.969) | OK | ^[27]^ |
| Li_3_Sn/LPSC/LNO  @NCM811 | Stable  (RT,1.73mA/cm^2^,100MPa,  1.8 mAh/cm^2^, >300 cls) | Stable  (i_p_/i_v_=0.856) | OK | ^[4]^ |
| Na_10_Sn_4_/Na_3_PS_4_/ Na_10_Sn_4_ | Stable  (RT,0.13mA/cm^2^,50MPa  0.13mAh/cm^2^, >100 cls) | Stable  (i_p_/i_v_=0.868) | OK | ^[28]^ |
| Na_15_Sn_4_/Na_3_SbS_4_/ Na_15_Sn_4_ | Stable  (RT,0.2mA/cm^2^,25MPa  0.2mAh/cm^2^, >100 cls) | Stable  (i_p_/i_v_=0.792) | OK | ^[29]^ |
| Na_9_Sn_4_/Na_3_SbS_4_/ Na_9_Sn_4_ | Stable  (RT,0.2mA/cm^2^,25MPa,  0.2mAh/cm^2^, >100 cls) | Stable  (i_p_/i_v_=0.780) | OK | ^[29]^ |

**References**

[1]	P. E. Blöchl, *Phys. Rev. B* **1994**, *50*, 17953.

[2]	J. P. Perdew, K. Burke, M. Ernzerhof, *Phys. Rev. Lett.* **1996**, *77*, 3865.

[3]	G. Henkelman, A. Arnaldsson, H. Jónsson, *Comput. Mater. Sci.* **2006**, *36*, 354.

[4]	S. Chen, Q. Cao, B. Tang, X. Yu, Z. Zhou, S.-H. Bo, Y. Guo, *ACS Energy Lett.* **2024**, *9*, 5373.

[5]	D. Lee, K.-H. Park, S. Y. Kim, J. Y. Jung, W. Lee, K. Kim, G. Jeong, J.-S. Yu, J. Choi, M.-S. Park, W. Cho, *J. Mater. Chem. A* **2021**, *9*, 17311.

[6]	D. Chatterjee, K. G. Naik, B. S. Vishnugopi, P. P. Mukherjee, *Adv. Sci.* **2023**, *11*, 2307455.

[7]	M. Chiku, W. Tsujiwaki, E. Higuchi, H. Inoue, *Electrochemistry* **2012**, *80*, 740.

[8]	S. Liu, L. Zhou, J. Han, K. Wen, S. Guan, C. Xue, Z. Zhang, B. Xu, Y. Lin, Y. Shen, L. Li, C.-W. Nan, *Adv. Energy Mater.* **2022**, *12*, 2200660.

[9]	A. Verma, H. Kawakami, H. Wada, A. Hirowatari, N. Ikeda, Y. Mizuno, T. Kotaka, K. Aotani, Y. Tabuchi, P. P. Mukherjee, *Cell Rep. Phys. Sci.* **2021**, *2*, 100301.

[10]	P. Barai, K. Higa, V. Srinivasan, *Phys. Chem. Chem. Phys.* **2017**, *19*, 20493.

[11]	Y. Li, S. Song, H. Kim, K. Nomoto, H. Kim, X. Sun, S. Hori, K. Suzuki, N. Matsui, M. Hirayama, T. Mizoguchi, T. Saito, T. Kamiyama, R. Kanno, *Science* **2023**, *381*, 50.

[12]	Y. Tanaka, K. Ueno, K. Mizuno, K. Takeuchi, T. Asano, A. Sakai, *Angew. Chem. Int. Ed.* **2023**, *62*, e202217581.

[13]	W. Xia, Y. Zhao, F. Zhao, K. Adair, R. Zhao, S. Li, R. Zou, Y. Zhao, X. Sun, *Chem. Rev.* **2022**, *122*, 3763.

[14]	L. Zhu, Y. Wang, Y. Wu, W. Feng, Z. Liu, W. Tang, X. Wang, Y. Xia, *Adv. Funct. Mater.* **2022**, *32*, 2201136.

[15]	P. Molaiyan, S. E. Mailhiot, K. Voges, A. M. Kantola, T. Hu, P. Michalowski, A. Kwade, V.-V. Telkki, U. Lassi, *Mater. Des.* **2023**, *227*, 111690.

[16]	C. Guan, Y. Yang, R. Ouyang, H. Jing, J. Yan, G. Li, H. Duan, H. Zhu, *Energy Storage Mater.* **2023**, *62*, 102936.

[17]	A. Emly, E. Kioupakis, A. Van der Ven, *Chem. Mater.* **2013**, *25*, 4663.

[18]	E. G. Herbert, W. E. Tenhaeff, N. J. Dudney, G. M. Pharr, *Thin Solid Films* **2011**, *520*, 413.

[19]	R. Maniwa, M. Calpa, N. C. Rosero-Navarro, A. Miura, K. Tadanaga, *J. Mater. Chem. A* **2021**, *9*, 400.

[20]	X. Lu, A. Windmüller, D. Schmidt, S. Schöner, C.-L. Tsai, H. Kungl, X. Liao, Y. Chen, S. Yu, H. Tempel, R.-A. Eichel, *ACS Appl. Mater. Interfaces* **2023**, *15*, 34973.

[21]	B. Tang, Y. Zhao, Z. Wang, S. Chen, Y. Wu, Y. Tseng, L. Li, Y. Guo, Z. Zhou, S.-H. Bo, *eScience* **2021**, *1*, 194.

[22]	K. Larson, E. A. Carmona, P. Albertus, *ACS Appl. Mater. Interfaces* **2023**, *15*, 49213.

[23]	F. Sagane, T. Abe, Z. Ogumi, *J. Power Sources* **2010**, *195*, 7466.

[24]	C. Hänsel, B. Singh, D. Kiwic, P. Canepa, D. Kundu, *Chem. Mater.* **2021**, *33*, 6029.

[25]	L. Zhao, W. Li, C. Wu, Q. Ai, L. Guo, Z. Chen, J. Zheng, M. Anderson, H. Guo, J. Lou, Y. Liang, Z. Fan, J. Zhu, Y. Yao, *Adv. Energy Mater.* **2023**, *13*, 2300679.

[26]	Y. Huang, B. Shao, F. Han, *J. Mater. Chem. A* **2022**, *10*, 12350.

[27]	Y. Huang, S. Chen, Y. Yang, Z.-T. Sun, X. Yu, C. Guan, R. Ouyang, Y. Guo, S.-H. Bo, H. Zhu, *Energy Mater. Adv.* **2024**, *5*, 0108.

[28]	N. Tanibata, K. Matsunoshita, H. Takeuchi, S. Akatsuka, M. Koga, H. Takeda, M. Nakayama, *J. Mater. Chem. A* **2023**, *11*, 25859.

[29]	Y. Huang, S. Chen, X. Yu, J. Zhou, C. Su, Y. Fu, Y. Guo, S.-H. Bo, H. Zhu, *Nano Energy* **2025**, *142*, 111227.
